# Meetings of Societies

**Published:** 1936

**Authors:** 


### Meetings of Societies

### Bristol Medico-Chirurgical Society

THE Annual Meeting of the Society was held on Wednesday, 16th October, at the University.

The President, Mr. Iles, was in the Chair, and ninety-four members were present.

Mr. Iles resigned his Chair to his successor, Dr. Richard Clarke, who gave his inaugural address entitled: **"The Evolution of a Casualty Clearing Station on the Western Front."**

Professor Hey Groves and Dr. Orr-Ewing seconded a vote of thanks, which was enthusiastically carried.

### BRISTOL MEDICO-CHIRURGICAL SOCIETY.

### INCOME AND EXPENDITURE ACCOUNT for the year ended 30th September, 1936.

### INCOME.

| | £ | s. | d. | £ | s. | d. |
|---|---|---|---|---|---|---|
| Members' Subscriptions .. | 372 | 4 | 6 | | | |
| Do., outstanding for 1935–1936 .. .. .. .. .. | 33 | 1 | 6 | | | |
| | | | | 405 | 6 | 0 |
| Interest on Bank Deposits .. .. | | | | 2 | 9 | 11 |
| Interest on £500 4 per cent. Consolidated Stock, less tax .. .. | | | | 15 | 7 | 6 |
| | | | | £423 | 3 | 5 |

### EXPENDITURE.

| | £ | s. | d. | £ | s. | d. |
|---|---|---|---|---|---|---|
| Grant to *The Bristol Medico-Chirurgical Journal* .. .. .. .. | | | | 112 | 5 | 3 |
| Librarian—Honorarium .. .. .. .. | | | | 15 | 0 | 0 |
| Miscellaneous Expenses— | | | | | | |
| Goodway–Refreshments | 16 | 16 | 0 | | | |
| Postages and Sundries | 12 | 8 | 3 | | | |
| Printing and Stationery | 9 | 0 | 6 | | | |
| Audit Fee .. .. .. .. | 2 | 2 | 0 | | | |
| Lantern and Cinematograph at Meetings .. | 5 | 5 | 0 | | | |
| University Marshal .. | 1 | 0 | 0 | | | |
| Lewis's Medical and Scientific Library— Subscription £5 10s. 0d.; Postages .. £0 15s. 8d. | 6 | 5 | 8 | | | |
| | | | | 52 | 17 | 5 |
| Balance, being excess of Income over Expenditure for the year, carried to Balance Sheet .. .. | | | | 243 | 0 | 9 |
| | | | | £423 | 3 | 5 |

### BALANCE SHEET as at 30th September, 1936.

### LIABILITIES AND CAPITAL.

| | £ | s. | d. | £ | s. | d. |
|---|---|---|---|---|---|---|
| Sundry Creditors .. .. | | | | 11 | 0 | 9 |
| Capital as at 30th Sept., 1935 .. .. .. .. .. | 889 | 16 | 6 | | | |
| *Add* Surplus for the year on Income and Expenditure Account .. .. | 243 | 0 | 9 | | | |
| | | | | 1,132 | 17 | 3 |
| | | | | £1,143 | 18 | 0 |

### ASSETS.

| | £ | s. | d. | £ | s. | d. |
|---|---|---|---|---|---|---|
| Cash at Bank— | | | | | | |
| Current Account .. .. | 502 | 5 | 2 | | | |
| Deposit Account .. .. | 171 | 2 | 4 | | | |
| | | | | 673 | 7 | 6 |
| £500 4 per cent. Consolidated Stock at cost .. .. .. .. .. .. | | | | 437 | 9 | 0 |
| Sundry Debtors— Members' Subscriptions for 1935–1936 outstanding .. | | | | 33 | 1 | 6 |
| | | | | £1,143 | 18 | 0 |

Audited and found correct,

H. E. KEELER,

*Chartered Accountant,*

*Auditor.*

16–18 Clare Street, Bristol 1.

*5th October*, 1936.

The annual reports of the Hon. Secretary, Hon. Treasurer and Hon. Librarian were read.

Mr. Clifford Moore was elected President-Elect; Mr. Cooke was re-elected Hon. Secretary; Mr. Iles, Hon. Treasurer; and Dr. George Parker, Hon. Medical Librarian.

---

The second meeting of the session was held on Wednesday, 11th November, at the University.

The President, Dr. Richard Clarke, was in the Chair, and eighty-six members were present.

Dr. Bergin showed skiagrams, and Dr. Taylor showed pathological specimens.

In their capacities as Professors of Surgery and Medicine, Mr. Rendle Short and Dr. Bruce Perry were elected Assistants to the Hon. Medical Librarian.

Dr. Clarke then introduced Dr. J. M. Woodburn Morrison, Professor of Radiology in the University of London, who gave an extraordinarily clear and interesting paper on "**X-ray Therapy.**"

A vote of thanks was proposed by Dr. Bergin and seconded by Dr. Bush. The appreciation of the audience was shown by long applause.

A short discussion followed, in which Mr. Moore and Mr. Wright took part, and Professor Morrison replied.

---

The third meeting of the session was held on Wednesday, 9th December, at the University.

The President, Dr. Richard Clarke, was in the Chair, and seventy members were present.

Dr. Frazer showed pathological specimens, and Dr. Bush showed skiagrams.

Professor S. E. Whitnall then gave a paper entitled: "**An Anatomist's View of Chiropractic.**"

Mr. Hey Groves, Mr. E. Watson-Williams and Dr. Orr-Ewing asked questions which were answered by Professor Whitnall.

---

The fourth meeting of the session was held on Wednesday, 13th January, at the University.

The President, Dr. Richard Clarke, was in the Chair, and fifty-three members were present.

Dr. Price showed pathological specimens, and Dr. Bergin showed skiagrams.

Mr. Wilfrid Adams gave his paper on "**Spinal Anæsthesia,**" opening the discussion in which joined Mr. Rendle Short, Mr. Kindersley, Mr. Ferrier Walters, Dr. Corfield, Mr. Paul, Mr. E. Watson-Williams, Dr. Leonard Moore and Mr. Cooke. Mr. Adams replied to many questions.

